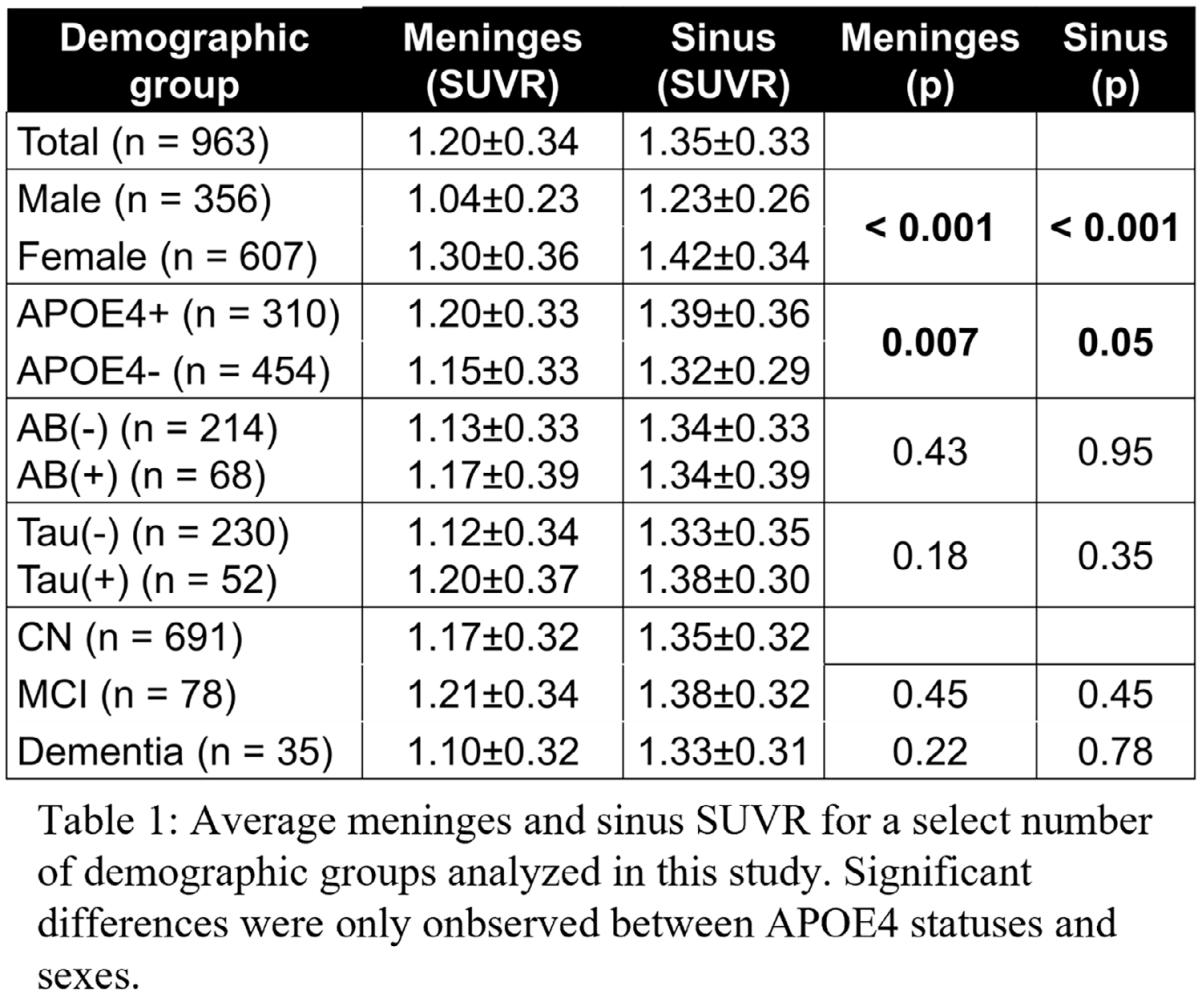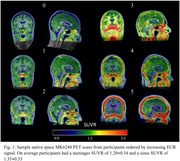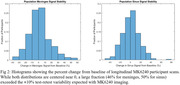# Longitudinal stability of extra‐cerebral off‐target binding in [F‐18]MK6240 PET images

**DOI:** 10.1002/alz.089808

**Published:** 2025-01-09

**Authors:** Andrew K McVea, Alexandra H DiFilippo, Max McLachlan, Brecca Bettcher, Sterling C. Johnson, Tobey J. Betthauser, Bradley T. Christian

**Affiliations:** ^1^ University of Wisconsin ‐ Madison, Madison, WI USA; ^2^ University of Wisconsin School of Medicine and Public Health, Madison, WI USA; ^3^ Waisman Center, University of Wisconsin‐Madison, Madison, WI USA; ^4^ Wisconsin Alzheimer's Disease Research Center, Madison, WI USA; ^5^ Wisconsin Alzheimer's Disease Research Center, School of Medicine and Public Health, University of Wisconsin‐Madison, Madison, WI USA; ^6^ Department of Medical Physics, University of Wisconsin‐Madison, Madison, WI USA

## Abstract

**Background:**

The spread of tau in Alzheimer’s Disease (AD) can be tracked in vivo using [F‐18]MK6240, a PET radioligand that binds to tau aggregates in AD with high affinity. However, significant MK6240 signal is also observed in the meninges and sinus and the extra cerebral binding (ECB) signal from these regions can spill into exterior brain regions complicating evaluation of early stage AD tauopathy. This study evaluates the magnitude and variability of ECB in a large imaging cohort to identify trends in this signal.

**Method:**

Participants included in this study were imaged at the University of Wisconsin–Madison on a Biograph mCT (n = 488) or ECAT HR+ (n = 475) PET scanner including 208 with longitudinal data taken an average of 2.0±1.0 years apart. Reconstructed PET images were smoothed using a 6mm^3^ gaussian kernel then processed using a standardized pipeline to generate SUVR images using the inferior cerebellar grey matter reference region. Meninges and sinus SUVR were compared across demographic groups of interest within the cohort and longitudinal changes in signal were quantified using the absolute change from baseline to follow up scans.

**Results:**

While there were not significant differences in ECB signal seen with age (p_meninges_= 0.89, p_sinus_= 0.98), amyloid (p_meninges_= 0.43, p_sinus_= 0.95) or tau statuses (p_meninges_= 0.18, p_sinus_= 0.35) a significantly higher average SUVR in the meninges and sinus for female (p_meninges_< 0.001, p_sinus_< 0.001) and APOE4+ participants (p_meninges_= 0.007, p_sinus_= 0.05) was observed. Longitudinally there was an average absolute SUVR difference between scans of 0.21±0.34 (12.1±10.9%) in the meninges and 0.24±0.33 (12.9±10.4%) in the sinus. This is more than triple the average longitudinal SUVR variability of 0.06±0.06 (6.5±6.0%) in the entorhinal region in our amyloid negative participants. APOE4+ participants additionally showed a significantly higher absolute change in the meninges (p = 0.05), however, the increase in the sinus was not significant (p = 0.59).

**Conclusions:**

MK6240 ECB can be highly variable and has the potential to bias tracer uptake outcomes. While we observed trends related to sex and genetic risk factors, further work to identify the underlying sources and correct for ECB is ongoing.